# Barriers to COVID-19 vaccination and strategies to increase trust and uptake among racial and ethnic minorities with HIV in South Florida: a qualitative study

**DOI:** 10.1186/s12889-025-25017-9

**Published:** 2025-11-10

**Authors:** Daisy Ramírez-Ortiz, Tatyana Moise, Seyedeh Yasaman Alemohammad, Mariolga Aymat, Aaliyah Gray, Michele Jean-Gilles, Diana M. Sheehan, Robert Ladner, Mary Jo Trepka

**Affiliations:** 1 Department of Epidemiology, Robert Stempel College of Public Health & Social Work, Florida International University, University Park, AHC 5, 11200 SW 8th Street, Miami, FL 33199 USA; 2 Department of Health Promotion and Disease Prevention, Robert Stempel College of Public Health & Social Work, Florida International University, Miami, FL USA; 3 Behavioral Science Research Corporation, Coral Gables, FL USA

**Keywords:** HIV, COVID-19, Vaccination, Racial and ethnic minority groups

## Abstract

**Background:**

People with HIV (PWH) from racial and ethnic minority groups in the United States (US) face a high risk for severe COVID-19 outcomes and have low uptake of the COVID-19 vaccine primary series and booster doses. This study aims to provide insights into barriers and facilitators to vaccination and identify strategies to increase vaccine trust and uptake in these populations.

**Methods:**

Between November and December 2022, we conducted qualitative interviews with 24 vaccinated and unvaccinated adult PWH who self-identified as Hispanic/Latinx, Black/African American, or Haitian, and were clients of the Miami-Dade County Ryan White HIV/AIDS Program in Florida, US. Data were analyzed using deductive thematic analysis.

**Results:**

Main barriers to vaccine uptake reported by participants included low or no perception of COVID-19 risk, concerns about safety and efficacy related to HIV status, mistrust of COVID-19 vaccines, general vaccine hesitancy, negative experiences and advice against vaccination within social networks, lack of provider recommendation, and exposure to negative messaging and misinformation about vaccines. Facilitators included perceived risk, awareness of the vaccines’ protective benefits for HIV-immunocompromised individuals and reducing transmission, encouragement and role modeling within social networks, provider recommendation, and exposure to accurate information from reputable sources. Some recommended strategies to increase uptake and trust in vaccines included incorporating vaccination into routine HIV care, leveraging peers with HIV, tailoring vaccine information and messaging, and partnering with trusted individuals for outreach.

**Conclusion:**

Vaccination efforts for PWH should prioritize addressing specific barriers and concerns related to their HIV status and tailoring strategies to meet their needs.

## Background

Vaccination has contributed to a substantial reduction in COVID-19 infections, hospitalizations, and deaths in the United States (US) and globally [1]. However, the COVID-19 virus continues to circulate, with new variants often emerging, posing a persistent risk to individual and public health [2, 3]. For people with HIV (PWH), the risks associated with COVID-19 infection remain a significant concern.

Compared to people without HIV, PWH—especially those with low CD4 cell counts, uncontrolled viral loads, advanced or untreated HIV, and comorbidities—face a higher risk of severe illness, hospitalization, and mortality from COVID-19 [4–9]. Even when viral suppression is achieved, immunodeficiency remains associated with an elevated risk of severe COVID-19 illness [10, 11]. PWH also have an increased risk of COVID-19 breakthrough infections compared to people without HIV or immune dysfunction [12–14], particularly if they have not received a booster dose [15]. Additionally, PWH from racial and ethnic minority groups experience worse COVID-19 outcomes [9, 16]. Hispanic and non-Hispanic Black PWH are more likely to be hospitalized or die from COVID-19 compared to non-Hispanic White PWH [9, 16]. Thus, PWH, regardless of CD4 cell count or viral load, should receive primary vaccination against COVID-19 and ongoing updated doses to prevent severe health outcomes and protect against emerging variants as recommended by the Centers for Disease Control and Prevention (CDC) [17].

The limited data on COVID-19 vaccination among PWH in the US shows that vaccine uptake varies widely in this population. US population-based data, state surveillance data, and smaller sample studies from May 2021 to March 2022 report uptake of a primary vaccine series among PWH ranging from 57% to 91% [18–21] and at least one vaccine dose ranging from 62% to 92% [18–20, 22]. One study using New York State surveillance data found that of the PWH who completed a primary vaccine series, 39% had received an additional primary or booster dose as of March 2022 [20]. Another study using data from eight healthcare organizations of the US Vaccine Safety Datalink Project reported that among PWH who completed a primary vaccine series by August 2021, 87% received an additional primary dose, and 24% received a booster dose by April 2022 [19]. In addition, a smaller study among racial and ethnic minority groups with HIV in Florida, including Hispanic, Black/African American, and Haitian individuals, reported that 84% of individuals had received a complete primary vaccine series as of March 2022, and 66% of these had received an additional primary or booster dose [21]. Studies reporting COVID-19 vaccination data by race/ethnicity show that the uptake of at least one vaccine dose, a primary series, and additional primary or booster doses is lower among Hispanic and non-Hispanic Black/African American PWH compared with non-Hispanic White PWH [18–22]. Increased uptake of COVID-19 vaccination among PWH, particularly for updated doses, is necessary.

Several factors have been reported to be associated with lower COVID-19 vaccination uptake among PWH, including lack of recent engagement in HIV care, low CD4 cell counts, detectable viral loads, and concerns about the vaccine’s impact on HIV disease progression or effectiveness of antiretroviral treatment [23]. Additional general factors that have been reported to be associated with lower COVID-19 vaccination uptake among PWH include vaccine hesitancy, misconceptions or conspiracies related to COVID-19 or vaccines, concerns about vaccine efficacy and safety (e.g., side effects), and access-related issues [21, 23]. In contrast, factors that have been positively associated with COVID-19 vaccination uptake among PWH include greater concern about susceptibility to COVID-19, confidence in the COVID-19 vaccines and their benefits, receiving positive messaging from trusted sources, recommendations from healthcare providers, a history of vaccination, and positive vaccination norms within their social networks [21, 23].

Nonetheless, limited research has provided a deeper understanding of factors related to the experience of living with HIV that influence vaccination decisions among PWH from racial and ethnic minority groups in the US [23]. Given the need to increase uptake of primary COVID-19 vaccination and ongoing updated doses among PWH, this study aims to understand barriers and facilitators to COVID-19 vaccine uptake and identify points of intervention among racial and ethnic minority groups with HIV in South Florida.

## Methods

In-depth semi-structured interviews were conducted between November and December 2022 among adults who were clients of the Miami-Dade County Ryan White HIV/AIDS Program (RWHAP). This US federal program provides no-cost HIV care, treatment, and support services to low-income PWH [24]. Participants were eligible for this study based on their previous participation in a survey conducted among RWHAP clients from January to March 2022, which examined factors associated with COVID-19 vaccination among PWH [21], and their agreement to be contacted for the qualitative phase. We purposively selected participants who identified as Hispanic or Latinx (hereafter Hispanic), non-Hispanic non-Haitian Black or African American (hereafter African American), and Haitian in the parent survey (*n* = 289) to focus on the experiences of racial and ethnic minorities with HIV. Non-Hispanic non-Haitian White participants were excluded (*n* = 10).

Our goal was to enroll eight participants per racial/ethnic group, with three fully vaccinated and five unvaccinated individuals. We intentionally oversampled unvaccinated participants to gain a deeper understanding of vaccination barriers. Vaccination status was initially determined from quantitative survey responses and confirmed during the verbal consent process. Participants who received all required doses of a primary vaccine series were classified as fully vaccinated; those who had not received any doses were classified as unvaccinated. A primary vaccine series was considered as two doses of a two-dose series (e.g., Pfizer or Moderna) or one dose of a single-dose vaccine (e.g., Johnson & Johnson). Nine participants who were partially vaccinated—having received only one dose of a two-dose series—were excluded from our sampling frame, resulting in 280 potential participants eligible for random sampling. To meet our sampling quotas, we generated six random lists of participant IDs, stratified by race/ethnicity and vaccination status. Each list included 8 potential fully vaccinated participants and 12 to 13 potential unvaccinated participants to contact. Only 12 unvaccinated Hispanic participants were available for inclusion in this group. Before reaching out, we verified that each selected individual had given their consent to be recontacted. We then proceeded down each list until quotas were met.

**Fig. 1:**
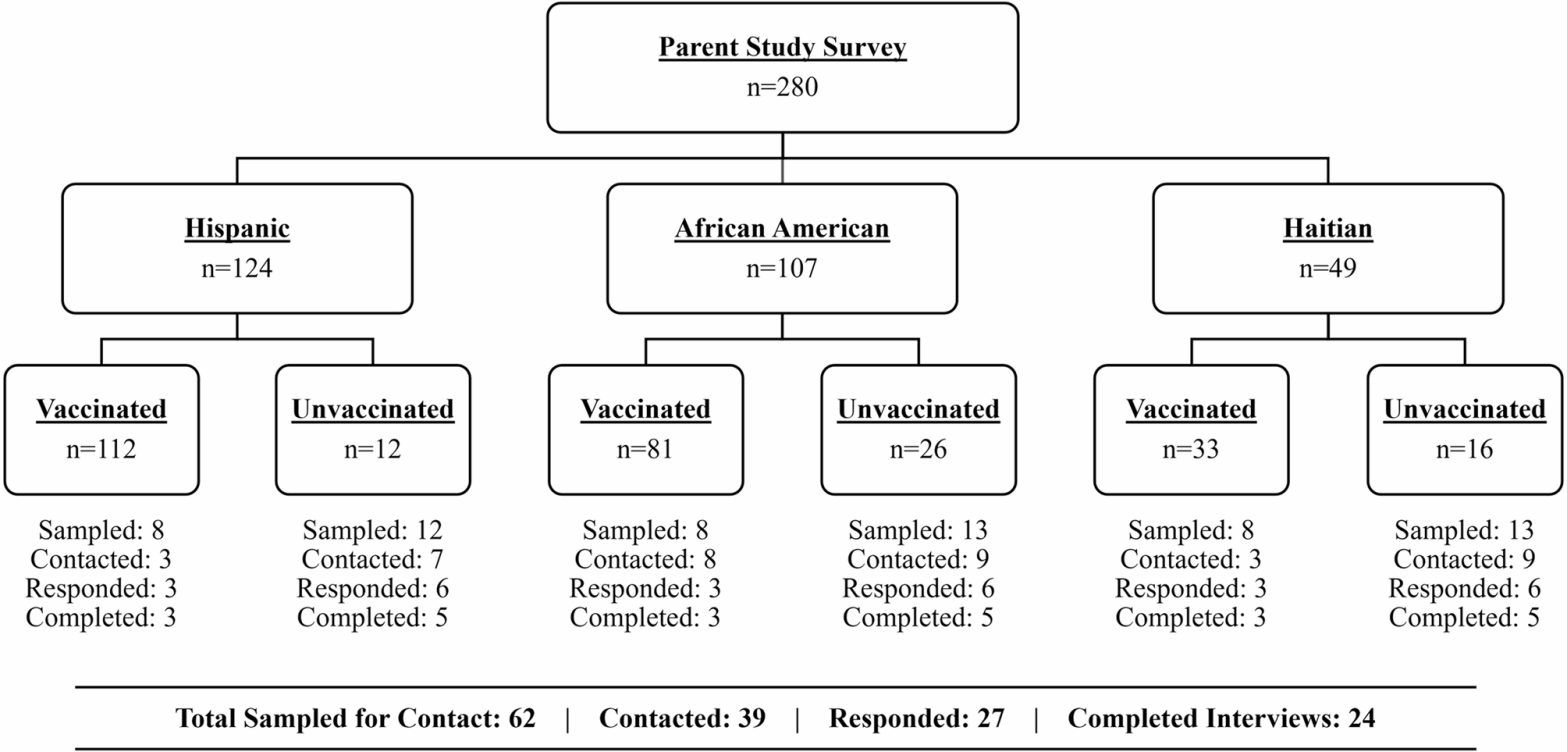
Flowchart showing the recruitment of qualitative interview participants by race/ethnicity and vaccination status. Six random lists were generated by race/ethnicity and vaccination status to sample individuals for contact. Hispanic = Hispanic or Latinx, African American = non-Hispanic non-Haitian Black or African American

We contacted participants via a standardized text message to introduce the study, followed by up to five call attempts across different days and times. Among the 280 survey respondents, 62 were included in the random sample lists, and 39 were contacted. Of those contacted, 12 did not respond to calls or texts or had inactive phone numbers. Of the 27 who responded, 24 completed a qualitative interview. Three others either asked to be called back, said they would call us back but did not, or were scheduled but we reached our target quota before the interview could take place. Figure 1 presents the recruitment and participation flowchart by race/ethnicity and vaccination status.

The semi-structured interview guide was developed based on factors identified in our previous survey study [21], which was informed by the Health Belief Model (HBM; [25]) and Socio-Ecological Model (SEM; [26]). We focused on key HBM constructs—perceived barriers (e.g., concerns about vaccine safety or efficacy), perceived benefits (e.g., protection from severe illness), and cues to action (e.g., informational influences)—as well as SEM’s emphasis on interpersonal context, particularly vaccination norms within social networks. The interview guide included three sections: one on perceived barriers and benefits, which asked about influences on vaccination decisions, concerns, and benefits regarding vaccines and booster shots for emerging variants; a second informed by both SEM (interpersonal context) and HBM (cues to action), which asked about how social network norms influenced participants’ decisions and the vaccine-related information they received and its sources; and a third on cues to action, which asked participants to suggest strategies to improve vaccine uptake and trust among PWH. Although the same questions were asked of both vaccinated and unvaccinated participants, we used two separate versions of the interview guide with wording to reflect vaccination status. Sample questions are provided in Table 1. Both interview guides were translated into Spanish and Haitian Creole by team members who are native speakers.

**Table 1:** Overview of interview guide sections, theoretical framework domains, and sample questions

Interview Section	Theoretical Framework Domain	Sample Questions
***A*.** Perceived Barriers and Benefits of COVID-19 Vaccination	HBM - Perceived Barriers and Perceived Benefits	What influenced your decision to get (not get) vaccinated with the COVID-19 vaccine?
B**.** COVID-19 Vaccination Social Norms and Information	SEM - Interpersonal Level; HBM - Cues to Action	Please tell me more about what your circle of friends and family did about the COVID-19 vaccine.; How did their decisions and opinions about the vaccine affect your decision to get (not get) the COVID-19 vaccine?
C. Strategies to Improve Uptake and Trust among PWH	HBM - Cues to Action	Please tell me what you think could be done to improve trust and acceptance of the COVID-19 vaccine among people with HIV.

Participants completed the interview over the phone in their preferred language: English, Spanish, or Haitian Creole. They received a $60 cash payment or gift card for groceries, based on their preference. Interviews lasted about 45 min or less and were audio recorded. Audio recordings were transcribed verbatim in the language in which the interview was conducted. Spanish and English interviews were first transcribed using NVivo Transcription [27] and then checked against the audio recordings for transcription fidelity and accuracy. Haitian Creole interviews were transcribed manually, as NVivo Transcription does not support this language. Spanish and Haitian Creole transcripts were translated into English by fully bilingual team members.

We used deductive thematic analysis for our data [28]. Two team members independently coded each transcript using NVivo 14 [29], working one transcript at a time. After coding each transcript, they met to compare codes, discuss discrepancies, and reach consensus before proceeding to the next transcript. If consensus was not reached, the two coders met with a third team member to discuss and collaboratively determine the final code. Data were initially coded into predefined constructs from the two theoretical models, and any additional data that emerged was considered. During analysis, we observed that responses related to the SEM domains—particularly social norms—as well as other content that emerged (e.g., perceptions of susceptibility and severity), aligned with HBM constructs. Therefore, we integrated codes into themes and organized them under the HBM framework to reduce redundancy in interpretation. The study was approved by Florida International University’s Institutional Review Board.

## Results

Participants had an average age of 53 years and self-identified as Hispanic, African American, or Haitian (Table 2). Most participants were female (63%), heterosexual (71%), college-educated (63%), and living above 100% of the US Federal Poverty Level (58%). Additionally, 63% were unvaccinated, and 38% completed the interview in English.

**Table 2:** Sociodemographic characteristics of participants (*n* = 24)

Characteristic	*n* (%)/mean (standard deviation)
Mean age (years)	53.3 (9.3) [range: 35–66]
Gender	
Cisgender female	15 (62.5)
Cisgender male	9 (37.5)
Sexual orientation	
Heterosexual	17 (70.8)
Lesbian, gay, bisexual	7 (29.2)
Ethnic and racial background ^a^	
Hispanic	8 (33.3)
African American	8 (33.3)
Haitian	8 (33.3)
Education	
Less than high school, high school diploma or General Educational Development (GED) certificate	9 (37.5)
Some college or college graduate	15 (62.5)
US federal poverty level	
≤ 100%	10 (41.7)
101–350%	14 (58.3)
Language of interview	
English	9 (37.5)
Spanish	7 (29.2)
Haitian Creole	8 (33.3)
Vaccination status	
Fully vaccinated^b^	9 (37.5)
Unvaccinated^c^	15 (62.5)

^a^ Hispanic = Hispanic or Latinx, African American = non-Hispanic non-Haitian Black or African American

^b^ Four fully vaccinated participants had received an additional primary or booster dose

^c^ Unvaccinated participants had not received any dose of the COVID-19 vaccine

The following section presents barriers and facilitators to COVID-19 vaccination uptake reported by participants, organized by constructs of the HBM. Recommended strategies to increase vaccine trust and uptake among PWH are also described. These findings are summarized in Table 3.

**Table 3:** Reported barriers and facilitators to COVID-19 vaccination categorized by Health Belief Model (HBM) constructs and recommended strategies to increase trust and uptake of vaccines among people with HIV (PWH)

HBM Construct	Barriers	Facilitators
Perceived COVID-19 Susceptibility and Severity	Low or no perceived risk of COVID-19 infection and severe complications	Perceived risk of COVID-19 infection and severe complications due to HIV immunocompromised status
Perceived Benefits of COVID-19 Vaccine Uptake	---	Awareness of vaccine protective benefits for HIV-immunocompromised and reducing transmission to others
Perceived Barriers to COVID-19 Vaccine Uptake	Concerns about safety and efficacy related to HIV-immunocompromised status Mistrust related to COVID-19 and vaccines General vaccine hesitancy Limited access when vaccines were first rolled out	---
Cues to COVID-19 Vaccine Uptake	Negative experiences and advice against vaccination within social networks Lack of recommendation from HIV care providers Exposure to negative messaging and misinformation about vaccines in media outlets	Positive encouragement and role modeling within social networks Recommendations from HIV care providers Exposure to accurate information about vaccines from reputable sources
Recommended Strategies		
HIV-specific	Open dialogue and recommendations from HIV care providers COVID-19 vaccine as part of recommended annual immunizations Sharing of case studies and peer experiences Distribution of vaccine information tailored for PWH	
General	Delivery of positive and encouraging messaging about vaccination Community outreach Vaccine reminders Incentives Single-dose vaccine	

### Perceived COVID-19 susceptibility and severity

 Vaccinated participants perceived themselves at risk of COVID-19 infection and severe complications due to compromised immune systems from HIV, comorbidities (e.g., diabetes, high blood pressure), and older age. These concerns prompted them to get the COVID-19 vaccine and boosters, as they believed vaccination would reduce their chances of experiencing serious illness, hospitalization, long-term complications, or death. Additionally, witnessing others get infected and suffer from severe complications, death, and long-COVID symptoms further heightened their risk perception and encouraged vaccination. In contrast, unvaccinated participants expressed low or no perceived risk of infection and severe complications, believing that having good health status, being adherent to their HIV medications, having an undetectable viral load, and implementing personal protective measures were sufficient for protection.

*“Well*,* I was immunocompromised [due to HIV]*,* so*,* that was the main factor [that influenced my decision to get vaccinated]. I want to protect myself as much as possible. … You have a better chance [if vaccinated] of coming out without having to be hospitalized and/or having any long-term effects to*,* you know? As opposed to not getting vaccinated at all.” - Fully vaccinated*,* 66 years old African American woman*.

*“… I guess I just figured*,* you know*,* I take all of my HIV medications*,* I’m undetectable… I felt I’m in good enough health. So*,* not that that made an excuse*,* but it’s just that … I just never did anything [about the COVID-19 vaccine]. ‘Cause I just figured… I keep my mask on*,* I wash my hands all the time*,* use hand sanitizer that… I guess I figured I was good [not getting vaccinated].” - Unvaccinated*,* 56 years old African American man*.

### Perceived benefits of COVID-19 vaccine uptake

 Vaccinated participants reported that their primary reasons for getting vaccinated and boosted were to protect themselves, particularly due to their HIV, and to protect others from COVID-19. They emphasized that vaccination contributes to preserving their health, preventing severe health consequences, and reducing transmission to others. Vaccinated participants also mentioned that getting boosted was essential for them to have enhanced protection against emerging variants. Notably, several unvaccinated participants acknowledged the benefits of COVID-19 vaccination in terms of providing health protection, controlling the spread of the virus, and reducing deaths. However, despite this awareness, they chose not to get vaccinated, while others perceived no benefits at all.

*“The reason why I got vaccinated was because I’m HIV and undetectable. … I was being cautious because of my HIV disease. …. I didn’t want to catch any disease. … I’m living in a complex … where there are a lot of elderly people*,* and they say the elderly are the main ones who might get it because their immune system… And I know my immune system was… with a virus itself. So*,* I was protecting myself and protecting those who was in my surroundings.” - Fully vaccinated*,* 65 years old African American woman*.

### Perceived barriers to COVID-19 vaccine uptake

 Several barriers were reported, including concerns about vaccine safety and efficacy, mistrust, hesitancy, and limited access. Both vaccinated and unvaccinated participants reported concerns about the safety and efficacy of COVID-19 vaccines, but these concerns did not deter vaccinated participants from getting the vaccine. Participants frequently mentioned concerns about experiencing common side effects, severe adverse events (e.g., seizures and death), and long-term effects (e.g., neurological and mobility issues) from vaccination. They believed they were particularly vulnerable to these potential negative effects from the vaccine because of their HIV immunocompromised status, CD4 count, and viral load levels. Additionally, concerns about potential vaccine effects were related to past negative reactions to the flu vaccine, a medical history of blood clots, and the perception that others were experiencing negative health events linked to the COVID-19 vaccines and getting sick more frequently after vaccination. Participants also expressed concerns about the rapid development and approval of the vaccine, the lack of adequate testing among PWH and in general, the vaccine’s components, and the potential for the vaccines themselves to cause COVID-19 infection and death. Several unvaccinated participants believed that they would be injected with a live virus, and that given their HIV immunocompromised status, this would make them sick or give them the COVID-19 disease. Participants also believed that certain vaccine brands were safer than others and that the safety of the vaccines depended on an individual’s body and health status. They also questioned the vaccines’ efficacy, expressing concerns about the number of doses required for adequate protection against COVID-19 and its variants, as well as the level of protection conferred against COVID-19, given that some vaccinated individuals still contracted the virus and sometimes experienced severe illness and death.

*“I was afraid that it would have side effects. And I think it was that. … Well*,* you always have the doubt about what*,* what they are injecting you. … Like my thinking is that many times in the vaccine*,* what they put you is the virus to make you immune. That was what really scared me*,* of me feeling healthy and that*,* and that it gives me a reaction … Yes*,* it’s really a little bit of fear of that*,* of my criteria [HIV]. … I was afraid that*,* that it gives*,* it gives me the [COVID-19] disease.” - Unvaccinated*,* 54 years old Hispanic woman*.

*“… my CD4 count wasn’t the best. So*,* I was afraid that if I was injected with the COVID-19 vaccine… hearing that there was a small portion of the actual virus in there*,* I was afraid that it was going to make me sick. Yeah. … Because our fear is the side effects [of the vaccine] and being that we [people with HIV] are immunocompromised that*,* that they can be a lot more damaging for us than others. So*,* the benefits to everyone else with a fully functioning healthy immune system isn’t the same in our side. … I had to make up in my own mind that … if my CD4 count was too low and my viral load was high… and then this*,* this [COVID-19] injection is like the flu vaccine*,* when people first started getting the flu vaccine. …. you get the flu for a minute*,* or you get sick from it. What if that same thing happened to us [PWH with the COVID-19 vaccine]?” - Unvaccinated*,* 38 years old African American man*.

Mistrust in the government, health agencies, and pharmaceuticals fueled vaccine hesitancy. Some unvaccinated participants described their mistrust towards the COVID-19 vaccines as being rooted in historical experimentation by the government, beliefs about COVID-19 being a common cold that previously existed, was deliberately planned by the government or health agencies, and was created by an international country, as well as concerns about the COVID-19 vaccines being driven by pharmaceutical profit interests and market competition. Unvaccinated participants also indicated that the rapid prioritization of the development of COVID-19 vaccines over improved treatments for other diseases such as HIV, the numerous vaccines and versions available, the government’s fluctuating information about vaccines and their level of protection and push for vaccination, and the conflict between politicians and health agencies over vaccines fueled their mistrust and hesitancy to get vaccinated.

*“It did make me think about the Tuskegee study. You know*,* people getting the vaccine. … I was gon’ go ahead and go through with it… at least for the first dosage*,* you know. And then when they came up with the second one*,* and then they had the booster*,* you know… now that’s where it kind of made me a little bit… I guess a little reluctant. … with me being African American and then that happened [the Tuskegee study] with African American men and then I*,* being a male… I just figured*,* you know*,* wow… I’m getting all this here shot up in me.” - Unvaccinated*,* 56 years old African American man*.

*“Well*,* the things that really frightened me the most is… I was seeing how so many different vaccines were popping up in such a short amount of time. So*,* it made me feel that either this was something that was already planned*,* and it was coordinated by the government or*,* you know*,* the World Health Organization or whoever was in charge. Or*,* that all of these different conglomerates- large medical companies were trying to find a vaccine so bad they could be… they could benefit from it financially. Either way*,* I didn’t feel confident about the different versions of a vaccine from different companies that were coming out.” - Unvaccinated*,* 38 years old African American man*.

Interestingly, two unvaccinated participants mentioned not following their healthcare provider’s recommendations to get the COVID-19 vaccine because they did not trust how their HIV treatment had been handled.

*“Well*,* my doctor always asked me to get the COVID vaccine and I never did. Even if he’s my primary doctor … because I know he’s part of this whole game [of multiple vaccine doses for COVID-19]*,* so simply*,* no. … because I have been on treatment… with HIV and they [providers] have changed my pills many times.… because they tell me that one [HIV pill] is better than another*,* but in the end … they are simply maintaining [the HIV] in their own way.” - Unvaccinated*,* 57 years old Hispanic man*.

*“I don’t trust even the whole HIV [treatment] thing with us. … I did not*,* I did not listen to my physician for the recommendation of getting it [the COVID-19 vaccine]. As I don’t follow a lot of the recommendations that [they] are always trying to push different drugs and new drugs on to you. … if I am healthy and on the [HIV] regimen that I’m on*,* then basically*,* I go by that.” - Unvaccinated*,* 64 years old Hispanic woman*.

Moreover, several unvaccinated participants expressed having no issues receiving other vaccines, such as those for flu and pneumonia, but lacked trust in the COVID-19 vaccines. Other unvaccinated participants also expressed being hesitant about vaccines in general, preferring natural immunity and the use of alternative medicine such as vitamins and teas over COVID-19 vaccination, and relying on their religious faith for protection.

*“My concerns… I don’t want to take it [the COVID-19 vaccine] and for something to happen to me. That’s the concern. … I don’t want it myself*,* no. Yes*,* I’m scared. I’m really scared of having other side effects … there are other vaccines that I get*,* they don’t do anything to me*,* you understand. It’s just like… when something can happen [if you get the COVID-19 vaccine]*,* you must be afraid…” - Unvaccinated*,* 49 years old Haitian woman*.

*“As long as I see in my own life*,* I won’t have anything [COVID-19]. … I always drink my little ginger*,* cinnamon… I am Haitian. … Truly when we have a problem*,* these are the little things we do. We make our little teas. Well*,* it’s these that I manage. I don’t manage vaccines.” - Unvaccinated*,* 61 years old Haitian woman*.

Only two participants reported difficulties accessing COVID-19 vaccines, citing limited vaccine availability and vaccination days at their HIV clinic, as well as long lines at public vaccination sites when the vaccines were first rolled out.

### Cues to COVID-19 vaccine uptake

 Social networks were reported as influencing vaccination decisions. Some vaccinated participants indicated that family members and friends served as role models, recommended vaccination, and provided motivation to get vaccinated. These vaccinated participants discussed COVID-19 vaccination with their family and friends to decide on the best course of action to protect themselves and their loved ones, considered the opinions of respected family members (e.g., elders, parents, siblings), and followed their advice to get vaccinated. On the contrary, negative influences within social networks—such as reports of vaccine side effects, perceived health issues caused by the vaccines, witnessing others survive COVID-19 without vaccination, and receiving advice against vaccination—discouraged some participants from getting vaccinated. Moreover, several unvaccinated participants acknowledged having conversations with family and friends, receiving advice to get vaccinated given their HIV status, receiving general encouragement, and reconsidering their decision after witnessing others in their social circle face severe consequences from COVID-19, yet they ultimately remained unvaccinated.

*“… [my family and friends influenced my decision to get vaccinated] because they were interested [in the COVID-19 vaccine]*,* and they interested me. And I also interested them so that they too could get it. … Oh yes [I talked to them about my decision to get vaccinated]. … they say I must protect my family. I must do right. ‘Cause*,* see*,* when someone is sick*,* they cannot see each other. And we are family*,* we cannot always be together. For our protection*,* we got the vaccine. And they justified that and got it too.” - Fully vaccinated*,* 53 years old Haitian woman*.

*“… when my sister got it [the vaccine]*,* it- it kind of made me think*,* Okay*,* well she got it. Maybe I should run out and get it too. … [she] sent me a picture*,* and I think she had- just like people have these voting stickers*,* “I voted!” I think she had sent me a picture saying*,* “I got the vaccine” or something. She had a sticker on*,* and she sent me the picture. And I was like*,* Wow! So*,* it kind of did motivate me a little bit. Cause*,* I was like*,* I like those voting stickers*,* you know*,* when I say I voted. And I was like … I should go and get me one just to show that I’ve got my vaccine. And then friends did it too. It was not just my sister. I had friends who actually did it and sent the pictures around. And they did the same thing for voting. “I voted.” “I- I got the vaccine.” So*,* it just kinda sparked that [motivation]”. - Unvaccinated*,* 56 years old African American man*.

Moreover, for some vaccinated participants, their HIV care providers were key in helping them trust the vaccine and decide to get vaccinated. They indicated that their HIV doctors and case managers had encouraging conversations with them about COVID-19 vaccination and recommended and offered it to them. On the other hand, some unvaccinated participants reported receiving vaccine recommendations and offers from their HIV care providers but did not follow them, while others did not receive any recommendations at all.

*“… I was convinced as quickly as… I knew that… the vaccine is good. It was my doctor or my case manager that I heard it [from]. … Yes [I talked to my doctor and case manager]. … it’s my doctor who asked me [about COVID-19 vaccination]. … My doctor proposed… it to me*,* and I told him that…*,* “yes*,* if it is something that is good for my health*,* I am ready for it.” - Fully vaccinated*,* 46 years old Haitian man*.

Participants also described how certain information they received about the COVID-19 vaccines and their sources influenced their decisions to get vaccinated or not. Vaccinated participants stated that they were informed about the vaccines’ efficacy in preventing severe consequences, though not necessarily infection, as well as the potential side effects and symptoms they could expect. They also received information about the higher susceptibility among chronically ill individuals, the high COVID-19 death rates, the emerging variants, the need for boosters to enhance protection, the positive safety results of vaccines, and the role of vaccination in reducing COVID-19 transmissions and protecting others. The main sources of information for vaccinated participants included television news channels, radio, the internet, social media platforms, vaccine developers, HIV care providers (i.e., doctors, nurses, case managers, clinics), health agencies and experts (i.e., CDC, NIH, Dr. Fauci, researchers), outdoor advertisements displayed throughout their community, and social networks. Some vaccinated participants expressed that witnessing the real-life negative impact of COVID-19 and the positive effects of vaccination, consulting with their HIV care providers, and receiving the information from health agencie s, experts, providers, and vaccine developers made the information they received trustworthy and counteracted the negative messaging circulating about vaccines (e.g., COVID-19 vaccines can cause death).

On the other hand, unvaccinated participants reported receiving negative messages mainly through television news channels, radio, newspapers, the internet, and social media platforms (i.e., Facebook, TikTok, YouTube). These messages emphasized the rushed development of the vaccines, the vaccines’ negative side effects, and the inability of the vaccines to prevent infection and death among vaccinated people. They also mentioned misinformation about the vaccines’ composition, such as claims that the vaccines contain a live virus, as well as the vaccines’ potential to cause death, including alleged cases of death among young athletes following vaccination. Additionally, one unvaccinated participant mentioned claims about people turning into wild beasts after vaccination and the vaccine being a bioweapon designed for global population control. However, some unvaccinated participants expressed that they received positive information about the vaccines and their benefits from their HIV care providers (i.e., doctors, case managers, clinics), health agencies and experts (i.e., CDC, Dr. Fauci), television and online news channels, radio, and through outdoor advertisements and events in their communities. Notably, several unvaccinated participants highlighted that the information about vaccines, booster doses, and variants was overwhelming, contradictory, and difficult for laypeople to understand, making it untrustworthy and discouraging them from getting vaccinated.

*“There was someone [on the internet] that said that when the person gets vaccinated*,* this is how the person becomes*,* this is how the person comes to look like. They show you a bunch of things on- on the internet. How the people come to look like- people who get vaccinated. That’s the reason which made me said I’d never get that vaccine even if I had- I had the means to travel*,* I preferred not to travel. I’ll never get the vaccine. Indeed*,* I never got it… and there’s nothing that’ll make me get vaccinated and I never got it. … Some said that if someone goes and gets the vaccine*,* they will die. Some said that [they] would be like a series of wild beasts. A bunch of words … that were running. I chose I won’t get it myself. If it is God’s will for me to die*,* I will die but I will not get the vaccine. It was a decision I made. … Oh yeah*,* yes. They [also] said a bunch of horrible words about the vaccine that made me not get it*,* yes. … they said … there are too many people in the world*,* they need to destroy some. That’s why they invented the vaccine.” - Unvaccinated*,* 52 years old Haitian woman*.

### Strategies to Increase Uptake and Trust in COVID-19 Vaccination among PWH

Participants recommended several HIV-specific and general ways to increase uptake and trust in COVID-19 vaccines among PWH. They emphasized the importance of informing and encouraging individuals while respecting their autonomy in making their own decisions about vaccination.

#### HIV-specific strategies

- *Open dialogue and recommendations from HIV care providers*: Participants recommended that HIV care providers, such as doctors, nurses, and case managers, maintain an open dialogue with their patients about COVID-19 and vaccination, and actively encourage and recommend vaccination, especially to those who are non-adherent to their care and treatment. However, they emphasized the importance of providers allowing patients to make their own decisions about vaccination at their own pace and without any pressure.
- *COVID-19 vaccine as part of recommended annual immunizations*: Participants also suggested that to increase uptake, the COVID-19 vaccine could be included as part of recommended annual immunizations.
- *Sharing of case studies and peer experiences*: Participants believed that sharing case studies demonstrating the positive impact and benefits of COVID-19 vaccination, as well as the consequences of being unvaccinated for PWH, could be an effective way to increase uptake. They also recommended leveraging peers with HIV to share their vaccination experiences to increase trust in vaccines.
- *Distribution of vaccine information tailored for PWH*: Participants recommended providing comprehensive information about COVID-19 vaccines, including their components, benefits, potential side effects, as well as the health risks associated with COVID-19 infection and the consequences of being unvaccinated. They also recommended sharing details about vaccination availability, locations, and procedures. Any information shared should be presented in non-medical, easy-to-understand language and specifically tailored for PWH. Participants emphasized the need to review and explain this information carefully to individuals to ensure understanding and facilitate informed decision-making about vaccination. The suggested formats for delivering this information include written materials (e.g., pamphlets, brochures, research reports), presentations (e.g., seminars, lectures, talks), small group discussions, one-on-one conversations or phone calls, and advertisements. Additionally, participants recommended disseminating information in the format of short texts, graphics, and videos via email, text messages, online websites, and social media platforms (e.g., Facebook, Instagram, TikTok), which they frequently access in their everyday lives. They highlighted that any personal communication and posts on social media platforms and online websites should come from sources they know and trust. Governmental health agencies (e.g., CDC, departments of health), researchers, pharmaceutical companies, health providers (e.g., doctors, case managers, clinics, hospitals), peers with HIV, outreach coordinators, community-based organizations and leaders, family members, and mainstream media (e.g., TV, radio, and newspapers) were identified as ideal messengers of this information.

#### General strategies

- *Delivery of positive and encouraging messaging about vaccination*: Participants suggested delivering positive and encouraging messages about vaccination to PWH through various outlets, including television news, radio, and social media, as well as displaying them in pharmacies, clinics, hospitals, and outdoor advertisements. They highlighted the importance of messaging about vaccine equality to improve trust in the vaccination process, emphasizing that everyone, regardless of socioeconomic status, receives the same vaccines and may access them at no cost.
- *Community outreach*: Participants suggested partnering with community-based organizations, religious institutions and leaders, and local businesses (e.g., barber shops, beauty salons, and laundromats) to initiate conversations and deliver messages about COVID-19 vaccination. They also suggested that HIV care providers and health agencies could conduct more outreach by making phone calls to individuals to discuss and offer COVID-19 vaccination.
- *Vaccine reminders*: Participants suggested that health providers and pharmacies could send emails or text messages to inform them about the availability of vaccines and remind them about booster shots.
- *Incentives*: Participants suggested offering monetary incentives to encourage vaccination.
- *Single-dose vaccine*: Participants mentioned that developing a COVID-19 vaccine that requires only a single dose and is highly effective in reducing COVID-19 transmission could significantly increase trust and acceptance.

## Discussion

This study provided insight into the general and HIV-specific barriers and facilitators influencing COVID-19 vaccination uptake among PWH from racial and ethnic minority groups. The main barriers to vaccine uptake reported included low or no perceived risk of COVID-19 infection and severe complications, concerns about safety and efficacy related to HIV status, mistrust related to COVID-19 and vaccines, general vaccine hesitancy, negative experiences and advice against vaccination within social networks, lack of provider recommendation, and exposure to negative messaging and misinformation about vaccines in media outlets. Facilitators included perceived risk of COVID-19 infection and severe complications, awareness of the vaccines’ protective benefits for HIV-immunocompromised individuals and reducing transmission to others, positive encouragement and role modeling within social networks, provider recommendations, and exposure to accurate information from reputable sources.

Low or no perceived risk of infection and severe complications was a barrier to vaccine uptake and mainly stemmed from the belief that having viral suppression and good health status was sufficient for protection. This barrier has been previously reported among PWH [23] and may have resulted from inadequate knowledge about COVID-19, exposure to information downplaying the risks for PWH, and confidence that they could avoid infection [30–32]. Additionally, as shown in a previous study [33], having strong immunological markers, such as an undetectable viral load, may lead PWH to believe they are in good health and therefore perceive their COVID-19 risk as low. However, findings from this study show that increased awareness and accurate risk perception can lead to vaccination, as participants who recognized their risk for COVID-19 severity due to their immunocompromised status chose to get vaccinated and boosted [23, 34]. Therefore, it is important to guide PWH in accurately understanding their actual COVID-19 risk, tailor any risk communication to the specific needs of PWH, and, as suggested by participants in this study, provide relatable case studies to modify their risk misperceptions.

Consistent with other studies among PWH [23], awareness of the protective benefits of vaccination and booster shots for HIV-immunocompromised individuals led to vaccination in this group. Additionally, the perceived benefit of protecting others and reducing transmission also encouraged vaccination within this group, which aligns with previous studies showing that a sense of collective responsibility can promote vaccination [23, 35]. However, despite recognizing these benefits, other concerns appeared to strongly influence decision-making among unvaccinated participants. Therefore, while promoting messaging about the protective benefits of COVID-19 vaccination and updated booster doses for both individuals and their communities is vital, it is also important to identify and address the specific concerns unvaccinated individuals may have.

Concerns about vaccine safety, such as side effects, severe adverse events, and long-term effects, were barriers to vaccination uptake, which align with previous studies among PWH [23, 36, 37]. These concerns were exacerbated by the limited safety data available for PWH and the belief that their immunocompromised status and uncontrolled viral load could increase their susceptibility to potential negative effects from the vaccine. Understandably, PWH may be especially concerned about the potential effects of COVID-19 vaccines due to their compromised immune function. However, clinical trials including immunocompromised individuals have demonstrated the safety of COVID-19 vaccines [38–41]. Additionally, while more comprehensive vaccine safety data is needed for PWH, available studies have shown that this group experienced only minor adverse events [42, 43], with an incidence similar to that of people without HIV [43–45]. CDC data on the safety of booster doses following a 3-dose primary series among immunocompromised individuals also show that while non-serious effects (e.g., injection site pain) were frequently reported, serious adverse events were rare in this group [46]. Thus, effectively disseminating available safety data to reassure PWH of vaccine safety and enhancing research specifically for this population is essential. It is also important to advise PWH about common expected side effects and address their individual concerns about vaccine effects, particularly since some participants in our study reported having a medical history of health conditions and reactions to other vaccines, which heightened their concerns. Another safety concern was the fear of being injected with a live virus vaccine and its potential to cause COVID-19 infection or sickness. This concern has been reported previously [47] and may arise from a lack of understanding about the vaccine components and the advice given to severely immunocompromised PWH to avoid live attenuated vaccines due to the risk of adverse reactions or disease [48]. This suggests the need to provide clear explanations about the components and mechanisms of the different types of vaccines recommended to PWH, including but not limited to COVID-19 vaccines, as concerns about containing a live virus might extend to other recommended vaccines (e.g., annual flu vaccine).

Furthermore, vaccine efficacy concerns were also cited as barriers to vaccination uptake, as participants questioned the level of protection conferred by vaccines and the need for multiple doses to ensure adequate protection. Concerns about vaccine efficacy have been previously reported among PWH [23] and may arise from a lack of understanding about the vaccines preventing severe illness but not necessarily infection, the potential for breakthrough infections due to reduced immune response over time and emerging variants, and the need for updated booster doses to sustain adequate protection. Additionally, data indicating a need for extra doses to achieve a robust immune response among PWH compared to those who are not immunocompromised [45, 49], and a reduced immune response among those with uncontrolled HIV [44, 45], might further contribute to efficacy concerns. Nonetheless, it is important to communicate that PWH generally have an adequate immune response when fully vaccinated [42, 44, 45, 49], while also being transparent about uncertainty and the evolving efficacy data [50]. More research on vaccine efficacy and durability of protection among PWH, particularly among those with uncontrolled HIV, is needed to increase vaccine confidence. Additionally, it is important to clarify to PWH that breakthrough infections could still occur after vaccination and that updated booster doses are required for added protection and do not necessarily reflect a failure of the vaccine’s primary series.

Mistrust and vaccine hesitancy were also barriers to vaccination uptake in this group. Mistrust towards COVID-19 and vaccines has been previously reported among PWH [23, 37] and is high among PWH from racial and ethnic minority groups [51]. In this sample, mistrust arose from historical issues, speculations about the origin of the COVID-19 virus, the rapid approval and prioritization of COVID-19 vaccines, the many vaccines released, doubts about pharmaceutical profit motives, and the inconsistent messaging and disputes surrounding these vaccines among different authorities, which align with previous studies [23, 51–55]. Although historically, PWH and racial and ethnic minority groups have reported mistrust towards public health authorities and pharmaceuticals for valid reasons [52, 56], the politicization of the COVID-19 response in the US, shifting recommendations, and widespread misinformation may have further exacerbated mistrust towards COVID-19 within this population [53, 54, 57]. Indeed, some unvaccinated participants reported trusting and receiving other vaccines but not those for COVID-19, underscoring the critical need to address mistrust specific to COVID-19 in this population. Efforts to address this barrier should focus on validating the historical roots of vaccine mistrust, addressing the specific beliefs and concerns PWH have about COVID-19 and vaccines, and involving trusted individuals to ensure more personalized and transparent conversations [51, 58]. Trusted individuals could include peers with HIV and community leaders, as suggested by participants. Moving forward, it is also crucial to improve stakeholder collaboration to avoid inconsistent messaging and ensure alignment on the most effective ways to communicate information about vaccines, including updated doses. Additionally, involving HIV care providers, particularly those from racial and ethnic minority communities [59, 60], is essential as they play a key role in mitigating medical mistrust and encouraging PWH to get vaccinated [51, 58]. Furthermore, as reported in other studies [23, 61], general vaccine hesitancy and preference for natural immunity and alternative medicine over vaccines also deterred vaccination in this group. Therefore, HIV care providers could also help address these barriers through personalized and culturally sensitive communication about vaccines and immunity [50].

Similar to previous studies among PWH [23, 37], while social networks served as cues for COVID-19 vaccination uptake through role modeling, recommendations, encouragement, and shared decisions, they also had a negative impact on vaccination decisions. Advice against vaccination, as well as the sharing of information within social networks about experienced vaccine side effects, health issues attributed to vaccination, and experiences of surviving COVID-19 without vaccination, led to non-vaccination in this group. Unfortunately, given that people tend to be clustered in social networks with homogenous attitudes and beliefs, unvaccinated PWH may remain hesitant to get vaccinated due to continuous reinforcement of their decisions within these networks [62]. However, research shows that even one vaccinated individual within a social network can positively influence vaccine uptake among others [62]. Therefore, it may be necessary to identify trusted and influential vaccine ambassadors within social networks who can respectfully challenge negative messaging and advice, provide accurate information, share their positive vaccination experiences, and encourage vaccination [58, 62]. This could include peers with HIV, trusted community members [58], as well as respected family members or friends, as reported in this study. Peers with HIV may be particularly effective, as they are more relatable, better positioned to share information and experiences tailored to the context of living with HIV, and were specifically suggested by participants in this study. Additionally, peers and other trusted individuals within social networks could use nudging strategies that leverage social norms, such as sharing photos with vaccination stickers, as described in this study, to further encourage PWH to get vaccinated. These strategies may help normalize vaccination, build trust, and promote vaccine uptake [63, 64].

Furthermore, as in other studies among PWH [23], cues from HIV care providers, such as conversations about the vaccines, recommendations, and offers, led to vaccination uptake. However, some participants reported either not receiving any recommendations at all or not following providers’ recommendations. It is important to note that in this study, negative experiences with how providers handled HIV treatment led to mistrust in their recommendations for COVID-19 vaccination. These findings suggest that more efforts are needed to enhance these recommendations and address concerns preventing PWH from acting on this advice. Therefore, it may be essential to integrate COVID-19 vaccination as a standard part of HIV care and annual immunizations. This approach would ensure routine conversations about COVID-19 vaccination and the active recommendation and offering of vaccination during care visits, as suggested by participants [58, 65]. It is also important that providers routinely implement patient-centered care practices such as open communication and shared decision-making [66], as these practices can promote trust and the uptake of other preventive strategies that are crucial to overall HIV care. As highlighted by PWH in this study, providers must respect the autonomy of PWH in deciding to get vaccinated. Additionally, as reflected in this study, providers’ efforts can be reinforced by case managers, who are trusted by PWH and may positively influence COVID-19 vaccination uptake [67].

Additionally, as reported in previous studies among PWH [23], negative messages and misinformation about vaccines discouraged vaccination in this group. These messages, which highlighted the negative side effects, the rapid approval process, and the vaccines’ limited ability to prevent infection, discouraged vaccination. Misinformation about vaccines causing infection and death, and conspiracy theories, even if they were implausible and far from believable, also deterred vaccination. In contrast, receiving clear and accurate information about COVID-19 and vaccines from reputable sources, along with consulting HIV care providers, appeared to counteract negative messages and encourage vaccination, in line with findings from other studies [23]. However, some PWH remained hesitant to get vaccinated even after receiving positive information from HIV care providers and other reputable sources. This hesitation is partly attributed to the perception of vaccine messaging as contradictory and complex to understand, which led to mistrust. These findings underscore the need to mitigate negative messages, debunk misinformation, and ensure the delivery of clear and consistent communication about vaccines. While engaging HIV care providers in addressing these barriers during care visits is essential [23, 51, 54], implementing creative strategies that leverage the sources and platforms of information used by unvaccinated individuals (e.g., social media) is also crucial. For example, HIV organizations and clinics could feature physicians and peers on their online and social media platforms to debunk misinformation and deliver positive messages tailored to PWH, similar to initiatives like “THE CONVERSATION: Between Us, About Us” from the Kaiser Family Foundation [58, 68]. Also, holding interactive webinars, Q&A sessions, social media live events, and podcasts to address common questions and concerns could be effective [58]. Moreover, based on participants’ suggestions and evidence-informed strategies [50], any communication effort about vaccines should prioritize presenting the information in a simple, clear, and tailored manner to enhance understanding, engaging known and trusted messengers, and leveraging diverse formats and daily accessible channels for dissemination (e.g., emails, texts, ads). It is also crucial to deliver consistent messaging across all sources to build trust in vaccines [50].

These reported barriers and facilitators were consistent across all racial and ethnic minority groups in this study, underscoring the usefulness of these findings for developing strategies to address common issues affecting COVID-19 vaccination among these groups. To increase uptake and trust in vaccines, PWH in this study supported the idea of incorporating COVID-19 vaccination into their routine HIV care and annual immunizations. As they highlighted, these efforts could be reinforced by providing case studies about the positive effects of vaccination on PWH and having peers share their experiences with vaccination. To supplement HIV-specific efforts, they also suggested distributing accurate and comprehensive vaccine information, sharing positive and encouraging messages about vaccines, partnering with community leaders and entities, conducting more direct outreach to PWH, and offering incentives. Although access was not a common issue in this sample, it is important to advertise ways to receive no-cost vaccination and share details about vaccination availability and locations to promote vaccine equity, which participants identified as important. Additionally, as mentioned by participants, clinics and pharmacies could automate vaccination reminders, particularly as updated doses will be needed for the long-term management of COVID-19 among PWH.

Our study has several limitations. First, our sample included adults who were receiving care through the Miami-Dade County Ryan White HIV/AIDS Program and residing in an urban setting predominantly composed of racial and ethnic minority groups. Therefore, experiences related to COVID-19 vaccination uptake may differ for PWH who are out of care or living in other US settings. Second, our data was collected during an active phase of the COVID-19 Public Health Emergency in the US, which may have influenced our findings. Exploring barriers to vaccination uptake among PWH as we continue to transition to routine management of the COVID-19 virus will be important.

## Conclusion

In conclusion, the barriers to COVID-19 vaccination reported by PWH in this study are understandable given historical mistrust, the novelty of COVID-19 and its vaccines, the handling of the pandemic response in the US, and the spread of misinformation. Tailored and adaptive strategies that consider the specific concerns of PWH, cues for vaccination, and information and communication needs identified in this study are warranted.

## Data Availability

The datasets generated and/or analyzed in the current study are not publicly available due to privacy concerns related to the qualitative nature of the data but are available from the corresponding author upon reasonable request.
